# Obesity inversely correlates with prostate-specific antigen levels in a population with normal screening results of prostate cancer in northwestern China

**DOI:** 10.1590/1414-431X20165272

**Published:** 2016-07-11

**Authors:** J. Zhang, M. Ma, X. Nan, B. Sheng

**Affiliations:** 1 Nutrition Department, The First Affiliated Hospital of Medical School, Xi'an Jiaotong University, Xi'an, China; 2 Geriatric Surgery Department, The First Affiliated Hospital of Medical School, Xi'an Jiaotong University, Xi'an, China; 3 Urology Institute, The First Affiliated Hospital of Medical School, Xi'an Jiaotong University, Xi'an, China

**Keywords:** Prostate-specific antigen, Body mass index, Obesity, Prostate cancer, Screening

## Abstract

Serum prostate-specific antigen (PSA) is a diagnostic biomarker of prostate cancer and is possibly associated with obesity. This study aimed to explore the relationships between obesity indicators [body mass index (BMI) and waist circumference (WC)] with PSA in Chinese men. A cross-sectional study of men aged 30-85 years undergoing prostate cancer screening was conducted from August 2008 to July 2013 in Xi'an, China. Data were obtained from clinical reports, condition was recorded based on self-report including demographics, weight, height, and WC (>90 cm=obese). Fasting blood glucose (FBG) and prostate volume (PV) were assessed clinically. Patients were grouped by BMI (normal=22.9, overweight=23-27.4, obese≥27.5 kg/m^2^). PSA parameters of density (PSAD), PSA serum level, and PSA increasing rate per year (PSAR) were calculated per BMI and age groups (30-40, 41-59, 60-85 years). Obesity indicators (BMI and WC) and PSA parameter relationships were modeled by age-stratified linear regression. Of 35,632 Chinese men surveyed, 13,084 were analyzed, including 13.44% obese, 57.44% overweight, and 29.12% normal weight, according to BMI; 25.84% were centrally (abdominally) obese according to WC. BMI and WC were negatively associated with all PSA parameters, except PSAD and PSAR [P<0.05, BMI: β=-0.081 (95%CI=-0.055 to -0.036), WC: β=-0.101 (-0.021 to -0.015)], and independent of FBG and PV (P<0.05) in an age-adjusted model. In conclusion, obesity was associated with lower PSA in Chinese men. Therefore, an individual's BMI and WC should be considered when PSA is used to screen for prostate cancer.

## Introduction

Prostate cancer is a leading cause of death among men and is the second most common cancer worldwide. In China, the incidence of prostate cancer has historically remained relatively low compared to global figures, estimated at about 33,000 cases per year, and anticipated to increase to >49,000 cases by 2020, with 20,000 deaths each year (1). The age-standardized incidence rate in the Chinese population has been estimated at 28.1 per 100,000, with an associated age-standardized death rate of 5.5 per 100,000 people (2). In Beijing, China, the incidence of prostate cancer increased from 55.3 per million in 2001 to 166.2 per million in 2010, with an alarming average annual growth rate of 9.2% (3).

Rapid economic development and industrialization have brought changes in traditional diets and increasingly sedentary lifestyles, resulting in an increasing prevalence of obesity and obesity-related chronic diseases in China over the past decade. Obesity rates have nearly tripled from 1991 (11.7%) to 2009 (29.2%) (4,5), based on a definition of obesity of people >27.5 kg/m^2^, as recommended by the World Health Organization (WHO) for the Asian population (6). Following this economic shift, the adult and pediatric patterns of obesity in China, as in other transitioning economies, have begun to more closely mirror the patterns observed in developed regions like the Unites States (4).

In addition to the effects of aging populations and increasing obesity rates, some studies have suggested that the increased prevalence of prostate cancer in the Chinese population might also be due to the wider implementation of improved, more sensitive prostate cancer screening in recent decades including the screening for serum prostate-specific antigen (PSA) (7). Recent studies across Asian populations have, however, demonstrated that PSA is age-dependent and increases significantly for every decade of life (8). It has also been reported that PSA levels are negatively associated with body mass index (BMI), primarily as result of a hemodilution effect caused by increased plasma volume (9,10). These relationships can affect the validity of PSA tests in prostate cancer screening, resulting in a high false positive rate in obese patients and some controversy over the use of PSA screening (11,12). Furthermore, the effects of age and obesity may be confounded by other related factors, such as fasting blood glucose (FBG) levels and prostate volume (PV), which have also been suggested to affect prostate cancer detection rates in Asian populations (13–16). Therefore, as the geriatric and obese populations are both increasing in China, there is an urgent need to better characterize the relationship between obesity and PSA parameters and to examine possible confounding effects in Chinese men undergoing prostate cancer screening.

Therefore, the objective of this study was to investigate the associations between obesity indicators [BMI and waist circumference (WC)] with FBG, PV, and PSA parameters [PSA serum level, PSA density (PSAD), and PSA increasing rate per year (PSAR)] in Chinese men undergoing physical exam routine screening, ultrasonography, and digital rectal examination (DRE). This study seeks to determine if obesity indicators and PSA parameters are independent predictors of detection rates.

## Material and Methods

### General study design

In this retrospective analysis, 35,632 Chinese men aged 30 to 85 years undergoing abdominal ultrasonography examination for prostate screening as part of their routine physical exam at the Affiliated Hospital of the Medical School at Xi'an Jiaotong University (Xi'an, China) from August 2008 to July 2014 were included. This study was approved by the Ethics Committee of the above-mentioned hospital, and was conducted in accordance with the Institutional Ethics Committee requirements. The need for individual consent was waived by the committee because of the retrospective nature of the study.

### Patient selection and data eligibility

All patients completed a survey at each visit, which is the routine practice at our institution. This survey helps to determine the life habits and risk factors for prostate cancer. Only data from the initial screening was considered for patients with multiple visits. Data were excluded from the analysis if: *i*) incomplete or missing survey responses were observed for required fields; *ii*) history of prostate cancer or suspected prostate cancer, benign prostatic hyperplasia (BPH), prostatitis, current active infection of the prostate, and/or inflammation with abnormal urinalysis; *iii*) the participant had undergone a DRE in the 7 days prior to screening or had undergone a cystoscopy or prostate needle biopsy within 1 month prior to screening; *iv*) the participant reported an age outside the 30-85 years window; *v*) the participant was taking prostate medication such as finasteride (Proscar^®^ or Propecia^®^), which have been reported to affect PSA (9), or *vi*) if the participant was observed to have FBG >15 mmol/L, BMI <17.5 kg/m^2^, and/or PSA >15 ng/mL.

### Data questionnaires

Data were collected from questionnaires containing questions about age, race, medication history, diabetes mellitus history, and prostate cancer history. Participants completed the questionnaires alone, and all responses were based on self-reported data. Measurement of height, weight, and WC were made at the hospital by a clinician during the medical examination. Age was validated using the identity card presented to the nurse.

### Determination of clinical parameters

In addition to completing the questionnaire, participants were asked to complete a screening panel including PSA, FBG, routine urinalysis, and DRE performed by a urologist at the site. Blood samples were collected after a 10-h fast from an antecubital vein. These samples were used to assay FBG and serum PSA prior to DRE. PSA analyses were done using the test Total PSA (Roche Diagnostics, Switzerland) on a Modular E-Modul System (Roche Diagnostics). FBG analyses were done using the glucose oxidase method from DiaSys Diagnostic Systems GmbH (Germany). All measurements were done in a central laboratory in a blind fashion, and according to the manufacturer's instructions. PV was determined by ultrasonography using the formula for an elliptic volume [(π/6)×height×width×length]. In the Xi'an area, transrectal ultrasound is not part of the routine screening tests for prostate cancer. PSA density was calculated as serum PSA (ng/mL) divided by PV (mL). PSAR was calculated as serum PSA (ng/mL) divided by age (years) (17).

### Determination of obesity factors

An HW-900Y ultrasonic wave height and weight scale (Jiangsu Hengfeng weighting, China) was used to find the height and weight of all patients. BMI was calculated as weight in kilograms divided by squared height in meters (kg/m^2^). WC was measured using a flexible, tension-sensitive, nonstretching tape measure placed directly on the skin. Participants stood relaxed, with arms folded comfortably across the chest so multiple WC measurements could more easily be made. Measures were made at the end of normal expiration with special attention paid to ensure the tape was positioned perpendicular to the long axis of the body and parallel to the floor. A series of four measurements were taken by a single, trained researcher from the right side at the following anatomical locations: *i*) superior border of the iliac crest, *ii*) midpoint between the iliac crest and the lowest rib, *iii*) umbilicus, and *iv*) minimal waist (18). Patients were grouped by BMI according to the WHO criteria for Asian populations (6), where patients with BMI <22.9 kg/m^2^ were considered in the normal range, 23-27.5 kg/m^2^ were considered overweight, and ≥27.5 kg/m^2^ were considered obese. Accordingly, abdominal (central) obesity was classified as WC >90 cm.

### Statistical analysis

Data were analyzed with SPSS 17.0 (IBM, USA) and reported as means±SD for continuous variables and frequencies (%) for categorical variables. Student's *t*-test and ANOVA were used to compare continuous variables and categorical variables, respectively. PSA levels were tested for normality and followed a normal distribution. Mean (±SD) PSA levels were calculated per BMI groups (normal range, overweight, and obese) and age groups (30-40, 41-59, and 60-85 years). Associations between obesity factors (BMI, WC) and PSA parameters (PSA level, PSAD, PSAR) were examined by linear regression models and stratified by age. As men with an elevated FBG (>110 mg/dL) have been shown to be more likely to have an enlarged prostate than men with normal FBG (15), this factor was also adjusted for in the linear regression model. A P value <0.05 was considered to be statistically significant.

## Results

### Characteristics of the men

**Figure 1:**
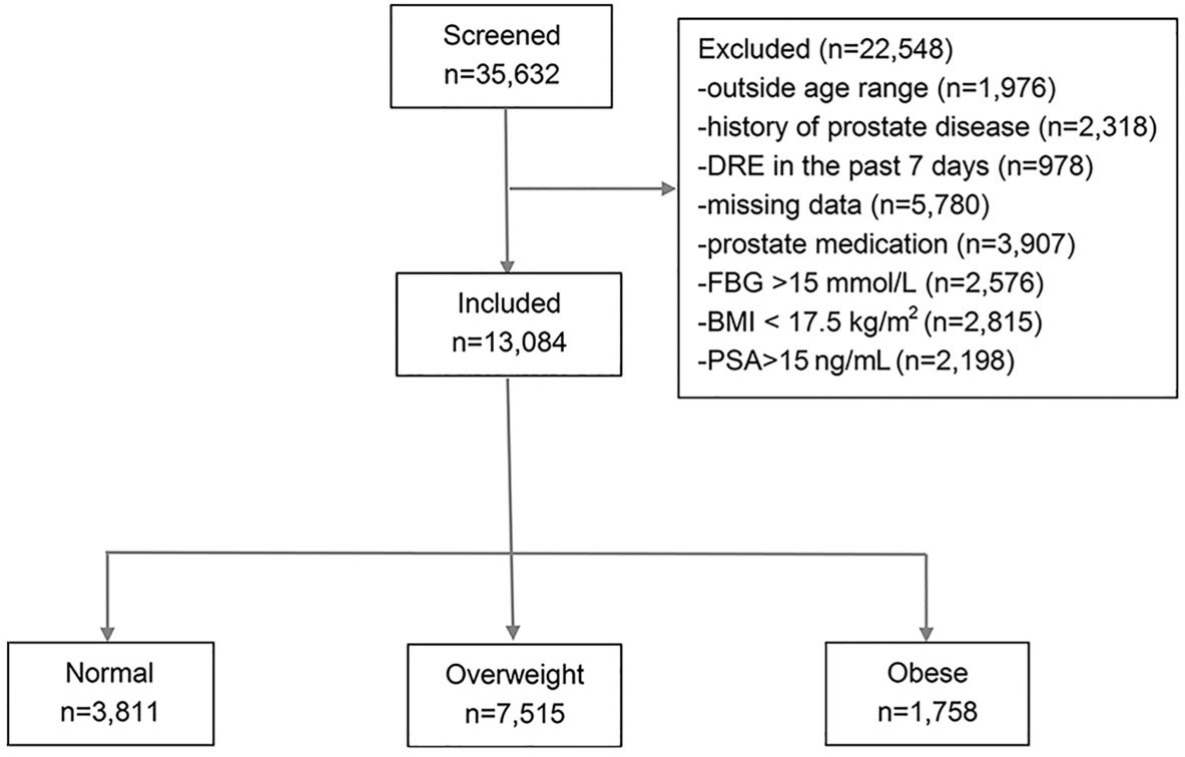
Flowchart of the included men.

Of the 35,632 Chinese men surveyed, 13,084 were included in the final analysis (Table 1 and Figure 1). Mean age was 54.2±13.7 (range 30 to 85) years; mean BMI was 24.5±2.9 kg/m^2^, and mean PSA was 2.05±1.63 ng/mL. Using BMI, participants were classified as 29.12% in the normal range (n=3811), 57.44% as overweight (n=7515), and 13.44% as obese (n=1758). Using WC, 25.84% (n=3381) of the participants were classified as being abdominally obese, and the remaining patients were classified as normal (n=9703). No significant differences in age were observed between BMI groups across age groups.

**Table:**
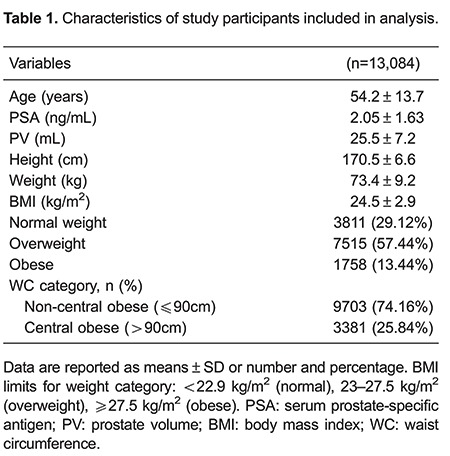

### BMI effects on PSA parameters and PV by age group

Mean age, FBG, PSA, PV, PSAD, and PSAR are shown for BMI groups (normal range, overweight, and obese) by age (≤40, 41-59, and 60-85 years) in Table 2. In all age groups, obese and overweight men (based on BMI) exhibited significantly lower serum PSA, PSAD, and PSAR than patients within the normal range (all P<0.05). Obese men also exhibited significantly higher PV and FBG levels (all P<0.005).

**Table:**
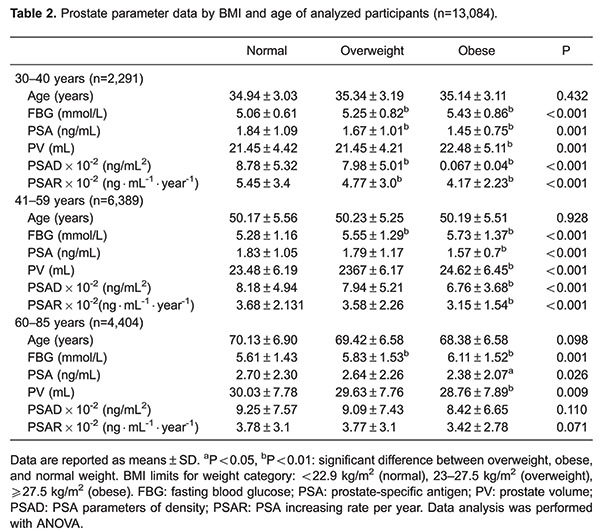

### WC effects on PSA parameters and PV by age group

Mean age, FBG, PSA, PV, PSAD, and PSAR are shown for normal *vs* abdominally obese by age in Table 3. There were no significant differences in age between the abdominally obese or normal patients by WC. For all age groups, abdominally obese men exhibited significantly lower serum PSA, PSAD, and PSAR (all P<0.05) compared with normal patients. For age groups 30-40 and 41-59, obese men also exhibited significantly higher FBG and PV levels (all P<0.01). In men aged 60-85, and compared to ≤40 and 41-59 significantly higher FBG levels were observed (P<0.001), but not PV.

**Table:**
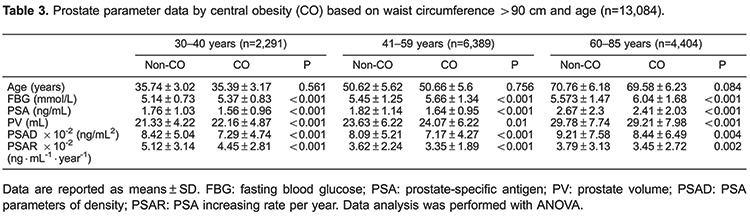

### Linear regression for obesity factors (BMI and WC) and PSA parameters, stratified by age

Linear regression results for BMI and PSA parameters was obtained and stratified by age, revealing that from ages ≤40 and 41-59, BMI was significantly associated with lower PSA, PSAD, and PSAR (all P<0.001), which remained significant when adjusted for FBG. Similarly, in men aged 60-85 years, BMI was significantly associated with lower PSA levels (P=0.032), and was found to be independent of FBG (P=0.021; Table 4). Linear regression revealed similar results for WC and PSA, stratified by age (Table 5).

**Table:**
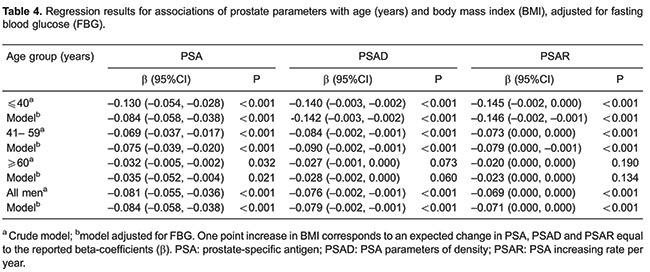

**Table:**
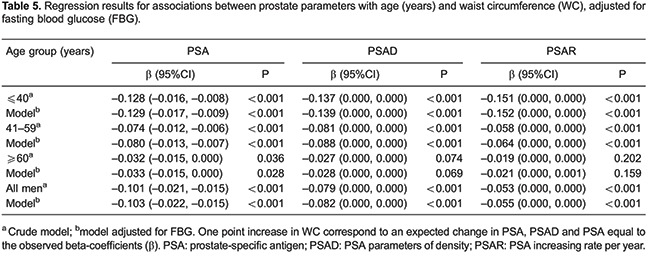

## Discussion

In a cross-sectional analysis of a large asymptomatic Chinese prostate screening population (n=13,084), it was observed that obese men exhibited consistently lower PSA parameters including PSA, PSAD, and PSAR. Furthermore, in all age groups surveyed from age 30 to 85 years, BMI and WC were inversely associated with serum PSA, PSAD, and PSAR levels independent of FBG and PV.

Although the relationship between obesity and PSA in prostate cancer remains controversial, previous studies have demonstrated an inverse relationship between serum PSA levels and obesity factors like BMI and WC in other populations of the world (8,19,20). Among 3000 healthy men examined in the San Antonio Center for Biomarkers of Risk of Prostate Carcinoma (SABOR) trial, it was reported that high BMI was associated with lower PSA levels after controlling for age and race (19). Similarly, two recent studies of Asian populations also reported an inverse association between BMI and PSA (8,20). Notably, the study conducted in a Korean population found an inverse association in men younger than 60 years, further suggesting possible effects of age and health on prostate cancer detection rates by PSA level. Compared to previous studies, the current study included a much larger sample size and was able to control for other factors including FBG and PV. In addition, the inverse relationship between BMI and WC, with PSA was clearly observed in Chinese men with age ranges from 30 to 85 years in this study.

Some researchers have suggested a hemodilution hypothesis for the association between BMI and PSA levels, which suggests that obesity increases plasma volume and hemodilution, thus reducing the circulating PSA levels (21). This hypothesis is based on the premise that blood PSA concentration is a function of plasma volume, as well as PSA expression and PSA leakage into the circulatory tract (22). Alternatively, another theory has been proposed whereby PSA is influenced by steroid hormone levels, referred to as the "steroid hormone metabolism hypothesis" (22). It is also highly likely that obesity influences PSA level through multiple pathways. Obesity could potentially alter the levels of multiple hormones and growth factors (e.g., testosterone, estrogen, leptin, insulin, and IGF-1) with competing effects on prostate growth and size (23). For obese individuals, a high amount of adipose tissue could improve aromatase activity to make cyclic estrogen levels increase (24). This result may be due to regulation via androgens and estradiol from adipose tissues.

In this study, inverse associations were observed between obesity factors and PSA, PSAD, and PSAR after adjustment for FBG. Simultaneously, this study also revealed the novel finding that abdominal obesity determined using WC may indeed have no appreciable effects on serum PSA levels after age adjustment. Furthermore, PSAD and PSAR levels were detectable with similar relationships in Chinese men older than 60 years undergoing prostate cancer screening, an age group well known for its high prostate cancer incidence. The presence of overweight or obesity should not influence the decision to perform PSA screening. Notably, these results confirm that screening PSA levels would not be unrealistic in this population.

Despite these encouraging results, it is important to note the limitations of these findings. In clinical practice, this significant negative correlation between obesity factors and PSA might be influenced by a number of factors present in diverse general screening populations. For instance, in a prospective cohort study of Caucasian men aged 40-79 years, serum PSA levels increased at a rate of 3.6% per year, with older men exhibiting more rapid increases in serum PSA compared to younger men, and men without diabetes exhibiting more rapid increases in serum PSA levels compared to men with diabetes (25). Hypertension, however, was not associated with alterations in serum PSA levels. Thus, prostate screening in the elderly may be subject to age effects but not specifically to obesity, which cannot be directly extrapolated to varied populations. Additionally, BPH and chronic urinary tract infection may affect serum PSA levels (25). In this present study, BMI and WC had weak effects on PV, and increases in PV may have resulted from BPH or prostatitis, which were excluded from this study. In obese men aged >60 years, a high frequency of untreated BPH might result from the limited access to treatment for some medical conditions in northwest China, which are related to the lack of early detection and prostate disease information for patients, long-term alcohol abuse, and chronic urinary tract infection that produce conflicting results in studies of obesity and BPH (26–28). As BPH is common in men >50 years old (about 60% of men aged >50 years have histological evidence of BPH, increasing to 80% after age 70 (29)), this study may be limited because these factors were not comprehensively tested, nor were results in Chinese populations compared directly to those of other regions. Another important consideration for these results is the use of different BMI values in Asian populations to define obesity than in Western populations (6). A lower cut-off BMI value of 27.5 is commonly used in Asian populations compared to the more common cut-off of 30. This is because adverse effects due to obesity are considered to occur at lower BMI in Asian populations, but this issue remains controversial (6). In addition, this study did not control for ethnicities, lifestyle, or functional sex hormone levels, which may play a role in prostate cancer development and detection, potentially altering PSA levels (30–33). Finally, only FBG was used as a marker for obesity-related diabetes because it is routinely measured and is inexpensive. Future studies could measure other markers such as insulin levels and IGF-1. Further large cohort studies are required in the future to test the consistency of these results between different populations and with consideration for other commonly encountered clinical factors. Despite these limitations, the large sample size, power of the results, and novel findings add knowledge to the current understanding of the relationships between obesity and PSA screening for prostate cancer. The relationship between obesity markers and other markers of prostate cancer should also be studied in the future.

In conclusion, this study revealed that obesity factors (BMI and WC) were associated with lower PSA levels in asymptomatic Chinese men aged 30 to 85 years undergoing routine prostate cancer screening, and these factors were independent of FBG and PV. When PSA is used to screen for prostate cancer, BMI must be taken into account to avoid misdiagnosis, especially in aged populations; however, when screening subjects older than 60 years, the PSA test may still be considered as an appropriate screening tool for prostate cancer in overweight and obese patients.
